# Young-Old City-Dwellers Outperform Village Counterparts in Attention and Verbal Control Tasks

**DOI:** 10.3389/fpsyg.2019.01224

**Published:** 2019-05-28

**Authors:** Hana Stepankova Georgi, Zuzana Frydrychova, Karolina Horakova Vlckova, Lucie Vidovicova, Zdenek Sulc, Jiri Lukavsky

**Affiliations:** National Institute of Mental Health, Klecany, Czechia

**Keywords:** urban, rural, environment, aging, cognition, attention, test norms

## Abstract

Cognitive performance is dynamic and shaped by individual biological and environmental factors throughout life. In psychology, besides the effects of age, education, and other often studied factors, the complexity of the lived-in environment and urbanicity in that context are yet to be elucidated. In this observational cross-sectional study, we compare cognitive performance in standard neuropsychological tests in healthy older persons from three different types of settlements in the Czechia: the capital city of Prague, towns, and villages. The groups were equal in terms of the age-band (60–74 years), the distribution of gender, education, past and current leisure activities, and cognitive health status (MMSE score). The results showed that Prague citizens had consistently better performance in all verbal tests (for memory and verbal control, i.e., executive function) and attention than persons from other areas. The groups did not differ in timed visuo-graphomotor performance. The conclusion is that the complex environment of a city may promote, in the long-term, certain cognitive abilities, distinguishable even in a developed, culturally homogenous country. The implications are: (a) the description of samples used in normative studies should include information on the lived-in environment for the reference of researchers and clinicians; and (b) individual clinical assessment should reflect the role of the patient’s environment where appropriate. The exact mechanisms and causes of the differences need further investigation.

## Introduction

Cognitive performance stems from biological predispositions (nature) and environmental and situational factors (nurture) (Kan et al., 2013; Tucker-Drob et al., 2013). It follows trajectories throughout life, gradually developing and improving, and eventually plateauing or experiencing decline and impairment. Cognitive trajectories have some features that are typical and some that are unique, with high inter-individual and intra-individual variability (Li et al., 2017). In general, slowing of psychomotor speed and reasoning, and lasting or even improving vocabulary/crystallized intelligence are observed during aging (Salthouse, 2019). In older age, the cognitive decline seems to be a natural phenomenon, just like functional somatic decline. A certain threshold of capabilities in both the domains, mental and physical, is key to personal functional independence. The threshold or a distance to it in the mental domain is assessed with neuropsychological tests (Weintraub et al., 2009; Arsenault-Lapierre et al., 2011; Litvan et al., 2012; Bondi et al., 2014). Cognitive performance in tests is known to correlate with age, education, and, in some tests, with gender (Beeri et al., 2006). Most tests are known to be culture-dependent (Rivera Mindt et al., 2010), i.e., a certain cultural background, usually associated with ethnicity, may promote or, on the other hand, hinder performance in some types of tests (Suzuki and Aronson, 2005). Thus, the performance has a relation to previous experience, which includes education and profession (Scarmeas and Stern, 2003; Kramer et al., 2004). Norms that are used to evaluate a person’s cognition should be adequate, i.e., if the normal performance is age-related, the norms should reflect age. The same applies to education and gender. In normative studies, this is acknowledged by the provision of regression equations with the relevant factors included, or tables of the scaled scores, percentiles, and other factors adjusted to age, education, or gender.

Besides these quite well-researched factors, there are others known to impair immediate performance in tests, such as pain (Moriarty et al., 2011) and sleep disturbances (Wennberg et al., 2017). Other factors are at play in long-term, both in their presence and effects on cognition. For example, the literature suggests a strong relationship between late-life depression, anxiety, and impaired cognition (Beaudreau and O’Hara, 2008; van den Kommer et al., 2013). Global perceived stress (Munoz et al., 2015) and especially chronic stress are other risk factors for cognitive performance in aging (Marin et al., 2011). Among late-life cognition-promoting factors, the literature points at marital status – not widowed, not divorced in midlife adulthood (Håkansson et al., 2009) and mentally or physically demanding leisure activities (Verghese et al., 2003; Frydrychova et al., 2018). Another potential positive factor may be social engagement, yet its role in cognitive maintenance remains unsolved (Weber, 2016). Sample descriptions, not only in normative studies, often include some of these characteristics.

Construction of norms requires large samples of healthy subjects. Normative studies of neuropsychological methods, timed and not timed, use subjects in predefined age groups, usually from one type of environment (Dufouil et al., 2000; Narazaki et al., 2013) or large cities where research institutes and universities are located (Crum et al., 1993; Machado et al., 2009). In general, it is not a rule that normative studies would state which type of settlements the subjects come from Tulsky et al. (2003), Beeri et al. (2006), and Weintraub et al. (2009), but some offer at least a vague description such as “different regions,” “central parts,” or “regional areas” (Tallberg, 2005; Aranciva et al., 2012; Senior et al., 2018), and only sometimes do we see “urban” or “rural” in the sample description (Ganguli et al., 1991; Piguet et al., 2001; Hashimoto et al., 2006; Costa et al., 2014; Türkes et al., 2015). Nevertheless, some studies acknowledge urban-rural differences in the context of cognition (Zhang et al., 2008; Miu et al., 2016; Nakamura et al., 2016; Lorenzo-López et al., 2017; Saenz et al., 2017; Zhao et al., 2017; Ren et al., 2018). Such studies usually originate in countries with quite distinctive discrepancies between urban and rural areas in terms of the density of population, socio-economic status, literacy rates, and availability of social-health care, among others – such as, for example, China, Mexico, Japan, Spain, and Ireland (Cassarino et al., 2016; Imai and Malaeb, 2016).

However, it is not known whether the differences in more sociologically homogenous countries affect cognitive performance. Potentially, this raises the threat of systematic assessment errors, e.g., building the norms based on the urban population may pose an inappropriately strict threshold when assessing people from rural areas. In the current study, we evaluate the differences between different areas of the Czech Republic (Czechia). Czechia is a good candidate to test this question, because it is a central European country and a member of the European Union, with 10.6 million inhabitants (average density of population 134 persons/km^2^), with an ethnically quite homogenous Caucasian population and almost no illiteracy (CZSO, 2011, 2014). There is only one city with a population exceeding one million inhabitants, and that is the capital city of Prague. Older persons perceive the pace of life of the capital city of Prague, other towns, and villages differently. The pace of life in Prague is the most hectic, while villages are the most tranquil according to their inhabitants. This is a common notion and it is shared by residents of each type of settlement we consider in this study (Frydrychova et al., 2017). However, there are not such profound differences in Czechia between urban, intermediate, and predominantly rural areas in terms of earnings, availability of health and social services, etc., as there are in India, China, and Brazil. The Czechia rural regions have only about 10% of people employed in agriculture, and the lifestyles are not “typically rural” as a result (Bernard et al., 2018). While people may differ in accumulated assets, in current generations of older people more than 90% are dependent on the nivellised old age pension as a sole income. The local analyses showed similar age-old pension, deaths per 1,000 population, average living floor area, availability of health, and social services (Pechrová and Šimpach, 2013; Chauvin et al., 2016). Also, a recent study found that there are no differences between urban and rural Czechia seniors in perceived loneliness or subjective poverty (Vidovicova, 2018). In Czechia, sociologists speak rather of a rural-urban continuum than a dichotomy (CZSO, 2014). The results of this study could serve as an estimate for differences in other European countries, which are culturally close.

In Czechia, norms for neuropsychological tests for older persons were supplied by the NANOK study – National Normative Study of Cognitive Determinants of Healthy Ageing (Štěpánková et al., 2015). For convenience reasons, psychometrists from 12 (out of 14) regions of Czechia were contacted and collected the data. Thus, the NANOK sample consisted of older adults living in various kinds of areas in terms of the level of urbanization in Czechia, including Prague. Secondary analysis of the NANOK data, which were primarily collected to provide a database for culture-relevant normative studies of neuropsychological tests, suggested that persons exposed to the demands of urban living in daily life during their productive adulthood perform better in speed-constrained tests when compared to gender-, age-, and education-pair-matched persons from other regions (Panenková et al., 2014; Horáková et al., 2016). The secondary analysis was performed on a small subsample of 84 Praguers aged 60–92 years and their pair-matched controls from other regions regardless of the exact type of settlement, i.e., non-Praguers were from settlements of various sizes. That led us to estimate that the shared environment of the busy city and proverbial hectic city life may, in the long term, promote attention and processing speed in older adults. The design of NANOK did not enable more detailed analysis.

Therefore, the current study, COURAGE (COgnition – URbanization – AGEing), is designed to illustrate potential differences between performance in timed cognitive tests of retired older persons in urban versus non-urban areas while considering the factors mentioned above that could moderate cognition. The practicality of duration of assessments limits the depth in which we can go in individual factors, however, the protocol offers a complex overview and outlook. The findings can have an impact on future normative studies. Also, it could inspire further research on an environmental role in the shaping of cognition in a lifelong perspective.

## Materials and Methods

### Participants

We recruited 366 retired, community-dwelling, young-old adults aged 60–74 years living in three types of settlements (Prague, towns, villages; see further in the text). Thus, we distinguished three groups in the sample accordingly. The inclusion criteria were: age; Czechia as mother tongue; retired and without paid work for a minimum of 2 years; having maintained the same type of place of living and work since the age of 40 (i.e., for a minimum of 20 years); no history of severe mental disorder (major depression, psychosis, substance abuse); no history of severe neurological disorder (brain infarction, traumatic brain injury, epilepsy, dementia); no chronic or acute pain affecting quality of life; no excessive daytime sleepiness; no presence of a non-compromised functional ability due to somatic disease or trauma; and no uncorrected sensory impairment. The inclusion criteria were listed in the recruiting materials and checked in the recruitment phase with specific questions. All participants signed informed consent forms in accordance with the Declaration of Helsinki. The study protocol was approved by the Institutional Ethical Review Board of the National Institute of Mental Health, Klecany, Czechia (under Nr. 61/16).

The recruitment was organized by the team members and trained psychometrists in the capital city of Prague and in towns and villages in Czechia, with an approximate target ratio of participants’ gender being 1:1, and with an approximate target ratio of education being lower: higher 1:1, in each type of settlement in 2017. Lower education refers to basic or lower secondary schools, such as trade schools, without the state leaving/graduation exam (i.e., without “maturita” in Czechia; 8–16 years of reported schooling in our sample); and higher education refers to secondary or high schools, including the state graduation exam, and tertiary schools or universities (11–33 years of reported schooling in our sample). This qualitative categorization of education is frequently used in normative studies of cognitive tests as the education system changed in the past century several times, and a number of years is not the best description of the level of acquired education (Bezdicek et al., 2015; Štěpánková et al., 2015; Kopecek et al., 2017; Nikolai et al., 2018). Advertising on the Internet, physicians’ and ophthalmologists’ offices, and snowball sampling were used. Universities of the Third Age (U3A) were not contacted, as their attendees do not represent a typical senior citizen. Residents of nursing homes were not included. All the persons were assessed by the psychometrists either at home or in the offices of the psychometrists according to each participant’s preference. The remuneration for the participation was 300 CZK (ca 12 €).

To avoid inclusion of data from persons with unrecognized mental health problems, we set ex-post inclusion criteria: the Mini-Mental State Examination (MMSE) score ≥ 26 (Folstein et al., 1975; Štěpánková et al., 2015); the Geriatric Depression Scale GDS15 < 7 (Sheikh and Yesavage, 1986); a negative clinical interview for depression; cognitive performance suggesting absence of cognitive disorder (Bondi et al., 2014); uncompromised instrumental activities of daily living (IADL) (Lawton and Brody, 1969, scoring according to Research Institute for Labour and Social Affairs CR); and good in-assessment conditions (current health status, undisturbed by unpredictable events, such as noise). In detail, the cognitive exclusion criteria were: (1) they had an impaired score, defined as > 1 SD below the age-corrected normative mean, on both measures within at least one cognitive domain (i.e., episodic memory, language, or speed/executive function); and (2) they had one impaired score, defined as > 1 SD below the age-corrected normative mean, in each of the three cognitive domains (Bondi et al., 2014).

The final sample after the application of all the exclusion criteria is described in detail in the Results section.

### Types of Settlement

For this study, we predefined three types of settlements: (1) the capital city of Prague, representing the largest city with a dense public transportation network, including a metro, with many seats of governmental bodies and tertiary education, and serving as the cultural and economic center of the region and the republic; (2) towns were mid-sized settlements with the status of a town and 20–50 thousand inhabitants. There are 46 towns of the kind in Czechia. Lastly, the third type of settlement was: (3) villages, defined as rural settlements with no more than 5000 inhabitants without the status of a town. There are 5445 villages of this type in Czechia.

The psychometrists were free to select from the pools of villages and towns designated in COURAGE according to accessibility. A detailed description of COURAGE settlements was published elsewhere (Frydrychova et al., 2017).

### Psychological Battery

COURAGE protocol included standard socio-demographic questions, as well as scales and questions on topics that could potentially influence current cognitive performance, such as GDS15 (Sheikh and Yesavage, 1986); IADL (Lawton and Brody, 1969); the life pace and environment rating (Frydrychova et al., 2017); leisure time activities (Frydrychova et al., 2018); and impediments, i.e., quality of life and cognition-impeding factors, including subjective health rating, psychopharmacological medication, and negative life events (Georgi et al., 2018); the Visual Analog Scale of Pain (VASP) (Scott and Huskisson, 1976); sleep disturbances (Rinaldi et al., 2001; Merlino et al., 2010); and stress-inducing household income and living conditions.

#### Cognitive Tests

Besides MMSE, COURAGE’s battery of psychological methods included standard cognitive tests, questionnaires, and a set of specific questions addressing the study goals and potential correlates. It was administered using paper and pencil, except for two computerized tests. The battery consisted of tests frequently used in research and clinical practice related to aging and age-related cognitive disorders (Beeri et al., 2006; Weintraub et al., 2009; Swindell et al., 2012; Nikolai et al., 2018), assessing different domains such as episodic memory, attention, (verbal and visuo-graphomotor) psychomotor speed, and executive functions.

##### Memory

###### Story

The Story used is a standardized version parallel to the Logical memory subtest of the Wechsler Memory Scale IIIa (Wechsler, 1997b; Frydrychová and Štěpánková, 2017), which is a measure of episodic memory and attention control, and also relies on intrinsic semantic organization. The dependent variable was the delayed recall of thematic units (0–7).

##### Visuo-graphomotor control

For the construct of visuo-graphomotor control, we selected three brief methods that are timed, require graphic production, and, to some extent, employ executive control.

###### Trail Making Test

In the Trail Making Test (TMT) part A (TMT-A), the task was to draw lines between digits in circles from 1 to 25 in ascending order as quickly as possible. In part B, TMT-B, the task was to draw lines connecting digits alternatively from 1 to 13, and letters from A to K, i.e., again between 25 targets. Both parts of the test were used. The times to complete successfully each part were the dependent variables, i.e., the time necessary for corrections is included in the score (Bezdicek et al., 2017). TMT is mainly a measure of visual search, mental tracking, motor speed, and set-shifting (Arbuthnott and Frank, 2000; Strauss, 2006; Lezak et al., 2012).

###### Digit Symbol Substitution Test

We used the standard form of the Digit Symbol Substitution Test (DSST) from the Wechsler Adult Intelligence Scale – III (Wechsler, 1997a). The task was to fill in as many boxes with graphic symbols belonging to numbers from 1 to 9 according to a key displayed on the top of the sheet. The dependent variable was the number of correctly drawn symbols in 120 s. DSST is a measure of visual search, processing speed, and memory (Joy et al., 2004).

##### Verbal control

For the construct of verbal control, we used several standard tests that require verbal production, are timed or prone to time passage, and employ executive control.

###### Prague Stroop Test

A variation of the Victoria Stroop Test (Troyer et al., 2006) was used: the Prague Stroop Test (PST) (Bezdicek et al., 2015). It comprises three subtests, each one featuring 6 rows and four columns (i.e., 24 in total) of stimuli in the same order of colors: dots (PST-D), neutral words (PST-W), and colors’ names printed in incongruent colors (PST-C). The task was to name the color in which the stimuli are printed as quickly as possible. Four basic colors are used in PST: red, green, blue, and yellow. The dependent variables are the times, in which all the items are named correctly, i.e., the time necessary for corrections was included in the score (Bezdicek et al., 2015). Stroop tests are used as measures of processing speed, executive control, inhibition, working memory, and attention control (Kane and Engle, 2003).

###### Category verbal fluency test – animals

The task (VF Animals) was to name as many unique words from a category of animals in 1 min as possible (Nikolai et al., 2018). The dependent variable was the number of animals named in 60 s. Category fluency is used as a measure of executive control, lexical access speed, and working memory (Shao et al., 2014; Mueller et al., 2015).

###### Rey auditory-verbal learning test – trial 1

The Rey Auditory-Verbal Learning Test (RAVLT) is one of the most-used tests of episodic memory (Lezak et al., 2012). The material is a list of 15 unrelated nouns (Frydrychová et al., 2018). In our study, we used only the Trial 1 (RAVLT1). The participants were asked to recall as many words as possible from the list, which was read once to him or her. RAVLT1 is a measure of attention, short-term, and working memory (Khosravi Fard et al., 2016).

##### Attention

A *Simple Reaction Time* task (SRT) and a *Go/No-Go task* (GNG) are included in the construct of attention in our study. SRT and GNG were presented on 7^′′^ Samsung SM-T280 tablets (Android 5.1) using the OpenSesame free platform (Mathôt et al., 2012) (SRT and GNG were programmed by JL). No operations with the tablets were required from the participants, other than tapping or not-tapping on the screen when they saw stimuli, which made it quite similar to paper-pencil methods. Thus, prior experience with ICT use was unnecessary. All participants tried a short demo version before each test. In SRT, a fixation cross appeared at the beginning of each trial. It disappeared, and, after a random interval (500–1000 ms), a red box appeared, and people were asked to touch the screen as quickly as possible. The stimulus was shown until the participant responded, but it was shown for no longer than 2000 ms. The SRT task included 5 practice trials and 50 experimental trials. The procedure and timing were analogous in GNG. Go stimuli were a green or blue box, and the no-go stimulus was a red box, visually presented at random latencies. The GNG task included 9 practice trials and 90 experimental trials. Both in SRT and GNG, the stimuli always appeared in the center of the tablet screen and had a fixed size of 22 mm. Both SRT and GNG are measures of attention, and psychomotor speed; GNG is also a measure of inhibition and executive control (Jones et al., 2016). The dependent variables were the mean reaction time for SRT and the mean reaction time to Go stimuli.

## Results

### Sample

Based on the ex-post criteria, we excluded 42 participants, and thus the final sample consisted of 324 persons (mean age 68.06 years, SD 3.08). The basic characteristics of the sample are in Table 1. We received 317 SRT and 313 GNG complete datasets from tablets. Due to reduced color sensitivity reported during the test, we had to omit one person from PST, SRT, and GNG. Final numbers of analyzed persons for each analysis are presented in Table 2.

**Table 1:** Sample characteristics and significance of differences between the groups using ANOVA or the chi-square test.

	Prague	Towns	Villages	*p*-value
**N**	**112**	**106**	**106**	
Age, years: M (SD)	68.73 (3.52)	68.42 (3.80)	66.99 (3.43)	<0.001
Women: n (%)	60 (54%)	54 (51%)	53 (50%)	n.s.
Education, years: M (SD)	14.07 (3.70)	13.64 (3.48)	13.04 (2.43)	n.s.
Lower education: n (%)	53 (47%)	53 (50%)	52 (50%)	n.s.
Manual profession: n (%)	45 (40%)	48 (46%)	50 (47%)	n.s.
Years in retirement: M (SD) Median [Min – Max]	8.40 (4.71) 7.50 [2–26]	9.38 (5.69) 8 [2–23]	8.42 (6.72) 7 [2–35]	n.s.
Having a partner: n (%)	84 (75%)	82 (77%)	90 (85%)	<0.01
Living alone: n (%)	33 (30%)	28 (26%)	10 (9%)	<0.001
Perceived life pace: M (SD) Median [Min – Max]	5.26 (1.59) 5 [2–8]	5.76 (1.52) 6 [2–8]	5.54 (1.70) 6 [2–8]	n.s.
Past activities: M (SD) Median [Min – Max]	5.34 (1.84) 5.5 [1–9]	5.39 (1.55) 5 [2–9]	5.39 (1.55) 6 [1–8]	n.s.
Current activities: M (SD) Median [Min – Max]	5.70 (1.54) 6 [1–9]	5.43 (1.43) 6 [1–8]	5.18 (1.49) 5 [1–9]	n.s.
GDS15: M (SD) Median [Min – Max]	1.90 (1.88) 1 [0–10]	2.04 (1.83) 2 [0–8]	1.60 (1.64) 1 [0–9]	n.s.
Impediments: M (SD) Median [Min – Max]	16.87 (3.42) 16 [10–25]	16.40 (3.55) 16 [10–28]	16.45 (3.44) 16 [10–25]	n.s.


*p-value = significance of the difference between the three groups (ANOVA, or Chi^2^ test for proportion variables); n.s. = non-significant, *p* > 0.1. Profession = manual or mental. Having a partner = irrespectively an official marital status. Living alone = living in an apartment or a house by oneself, without co-inhabitants. Perceived life pace (score 2–8; Likert-like scale, higher score for more hectic) = subjective level of time-demand at work and in life before retirement. Past = before retirement. Activities (score 0–9; yes/no for each) = number of regularly performed activities of attending a course at a University of the Third Age, attending other courses such as language or ICT, physical exercise, aerobic activity of medium intensity at least 2.5 h per week, hobby, using a computer, reading books, reading newspapers, and magazines, doing crossword puzzles or quizzes. GDS15: Geriatric Depression Scale -15. Impediments (score 7–35; Likert-like scale for each) = composed of subjective health status, current pain, sleeping problems, negative life events with emotional impact, mental health, anxiety for economic reasons, low-income objective problems (Georgi et al., 2018).*

**Table 2:** Associations between key characteristics and cognitive performance.

	N	Gender	Age	Education	Profession	Partner	Living	Pace	Activities past	Activities current	GDS15	Impediments
		**η^2^**	**r**	**η^2^**	**η^2^**	**η^2^**	**η^2^**	**r**	**r**	**r**	**r**	**r**

Story	324	0.009	0.052	0.052^***^	0.052^***^	0.007	0.007	0.009	0.089	0.242^***^	-0.101	0.062
TMT-A+	324	0.009	0.104	0.026^**^	0.038^***^	0.005	0.000	-0.073	-0.232^***^	-0.226^***^	0.114^*^	0.034
TMT-B+	324	0.006	0.229^***^	0.086^***^	0.092^***^	0.012	0.000	-0.146^**^	-0.264^***^	-0.213^***^	0.134^*^	0.032
DSST	324	0.043^***^	-0.188^***^	0.113^***^	0.119^***^	0.019^*^	0.001	0.100	0.332^***^	0.353^***^	-0.172^**^	0.029
CS Visuo-Grapho	324	0.023^**^	-0.207^***^	0.098^***^	0.111^***^	0.016^*^	0.000	0.126^*^	0.328^***^	0.314^***^	-0.166^**^	-0.015
PST-D	323	0.012^*^	0.164^**^	0.034^***^	0.007	0.000	0.000	-0.023	-0.232^***^	-0.206^***^	0.047	-0.059
PST-W	323	0.014^*^	0.206^***^	0.064^***^	0.023^**^	0.002	0.000	-0.043	-0.219^***^	-0.202^***^	0.044	0.008
PST-C+	323	0.006	0.187^***^	0.096^***^	0.066^***^	0.002	0.001	-0.041	-0.232^***^	-0.161^**^	0.088	0.034
VF Animals	324	0.002	-0.158^**^	0.017^*^	0.029^**^	0.005	0.006	0.021	0.245^***^	0.243^***^	-0.045	0.091
RAVLT1	324	0.042^***^	-0.061	0.072^***^	0.035^***^	0.007	0.003	0.034	0.096	0.288^***^	-0.119^*^	0.027
CS Verbal	323	0.025^**^	-0.222^***^	0.108^***^	0.059^***^	0.001	0.000	0.048	0.295^***^	0.316^***^	-0.099	0.038
SRT+	316	0.001	0.056	0.011	0.006	0.001	0.001	0.046	-0.089	-0.145^**^	0.027	0.019
GNG+	312	0.001	0.073	0.029^**^	0.019^*^	0.008	0.000	0.105	-0.128^*^	-0.141^*^	-0.030	0.002
CS Attention	312	0.001	-0.075	0.022^**^	0.014^*^	0.005	0.000	-0.075	0.111^*^	0.148^**^	0.010	-0.013


*Education = lower or higher. Profession = manual or mental. Partner = having a partner irrespectively an official marital status. Living = living in an apartment or a house by oneself, without co-inhabitants. Pace (score 2–8; Likert-like scale, higher score for more hectic) = subjective level of time-demand at work and in life before retirement. Past = before retirement. Activities (score 0–9; yes/no for each) = number of regularly performed activities. GDS15 = Geriatric Depression Scale – 15 score. Impediments (score 7–35; Likert-like scale for each) = composed of subjective health status, current pain, sleeping problems, negative life events with emotional impact, mental health, anxiety for economic reasons, low-income objective problems. r = Pearson correlation; η^2^ = eta squared – univariate ANOVA, effect size. CS, composite scores. + = scores, which were logarithmically transformed for the analyses (ln) and then back-transformed. ^∗^*p* < 0.05; ^∗∗^*p* < 0.01; ^∗∗∗^*p* ≤ 0.001.*

We compared the groups from Prague, towns, and villages to find possible differences in key characteristics that could, according to the literature, influence cognitive performance (Table 1), such as gender, age, level of education, type of profession in the past (manual/mental), living alone in or sharing a household, having a partner irrespective of official marital status or being currently without a partner, perceived pace of life in the past, past and current number of activities, score in GDS15, and impediments to well-being. All the analyses were based on chi-square tests for independent samples, and only age, the number of activities, GDS15, and impediments were analyzed with ANOVA.

We found that the groups did not differ in basic characteristics such as gender, education, type of profession, number of activities before retirement, and, at present, symptoms of depression and impediments related to their health and stress factors (Table 1). The Prague group was slightly older than the participants from other regions, and more citizens of Prague lived alone, i.e., without co-habitants. Those two parameters were analyzed as to their association with cognitive tests (cf. further and Table 2: age correlated significantly with cognitive performance, living alone did not. Therefore, only age was added as the covariate in ANCOVA).

We compared the proportions of specific leisure activities in different settlement types with χ^2^ test. We found that more Praguers reported computer-use (past and current), and attending to courses or to U3A (only currently) than did villagers and towners. On the other hand, participants from villages were more physically active (past and current) compared to other settlements. All differences were significant below *p* < 0.001.

### Cognitive Performance of the Sample

#### Data Preparation

First, cognitive data distribution was visually inspected. Due to skewness, TMT-A, TMT-B, PST-C, SRT, and GNG were submitted to logarithmic transformation [new score = ln (1 + raw score)] before further analyses. Log-transformed test scores were used for all analyses, and results were back-transformed [exp (new score) – 1] to the original scale. In SRT, we first removed all trials where participant did not respond. We dropped all trials with RT < 150 ms or above 3 SD of the individual. For each participant, we calculated the mean response time (RT). In GNG, we analyzed RT in go trials. We used the same filtering criteria (RT < 150 ms, RT > 3 SD). For each participant, we calculated the mean response time in go trials. The commission errors in GNG (responses to no-go trials) were excluded from the dataset (Mean = 1.1%, *SD* = 1.8, range 0–11.7). One-way ANOVA did not show a connection between settlement type and number of commission errors [*F*(2,310) = 0.28, *p* = 0.756].

#### Composite Scores

For clearer interpretation of the results, we created composite scores (CS) of the visuo-graphomotor control, verbal control, and attention domains by standardizing performance on each task and averaging the z-scores (cf. Festini et al., 2016). Construct reliability substantiated all three measures. Cronbach’s alpha was 0.80 for visuo-graphomotor control, 0.74 for verbal control, and 0.84 for attention.

### Cognition and Sociodemographic Factors

The relationship of cognitive performance to key characteristics (see below) was evaluated using parametric methods (ANOVA and Pearson correlation). Eta-squared and Pearson correlation coefficients (r) are presented in Table 2. In Tables, we report uncorrected *p*-values for multiple testing.

Performance in tests was correlated with several factors (Table 2), namely, gender, age, education, profession, and the number of activities previously and currently. Cognitive performance did not correlate with having a partner, living alone, perceived life pace, symptoms of depression, or impediments (negative life circumstances, health issues). Even within the selected age band (young-old adults), we found a correlation of age with performance in some tests. Because the settlement groups differed slightly in terms of age distribution, but not in other key characteristics (i.e., living alone), only age was added as a covariate in the concerned analyses.

For exploratory purposes, we investigated the associations between cognitive performance and engagement in specific leisure activities (past and current). We tested the association with Point-biserial correlation coefficient. Further, we report associations with *p* < 0.001. Computer use (past) was positively significantly associated with CS visuo-graphomotor control and CS verbal control; computer use (current) with Story, CS visuo-graphomotor control, and CS verbal control; doing crossword puzzles or quizzes (past) with CS visuo-graphomotor control; U3A (current) with Story, CS visuo-graphomotor control, and CS verbal control; non-U3A courses (current) with Story and RAVLT1; physical activity (past) with PST-D only. Other types of leisure activities were not significantly associated with cognitive performance.

### Cognitive Performance and the Type of Settlement

Differences in cognitive performance between three settlement groups (Prague, towns, and villages) were evaluated using ANOVA or ANCOVA (age as a covariate) for tests, which are significantly influenced by age (TMT-B, DSST, PST, and VF Animals, CS visuo-graphomotor control, and CS verbal control). We evaluated the association between having a partner, age and type of settlement, and performance in DSST and CS visuo-graphomotor control with ANCOVA. We did not find a significant association (*p* > 0.05). Therefore we do not report it further. We did not analyze specific activities with respect to cognition and settlement as our data did not provide enough information for detailed insight.

Subsequently, we performed Bayesian versions of ANOVA or ANCOVA and calculated Bayes Factors (BF) to evaluate the extent to which the observed results favor the null (or alternative) hypothesis (Quintana and Williams, 2018; Wagenmakers et al., 2018). BF analyses were performed using JASP (JASP Team, 2018), and all other analyses were performed using SPSS 23.0 (IBM Corp, 2015). The analyses showed support for the hypothesis of the different cognitive performance of citizens of Prague compared to residents of other regions. Bayes Factors showed extremely strong evidence for the difference in memory (Story), SRT, and CS attention. Strong evidence was found for PST-W, VF Animals, and GNG. We found strong evidence for the absence of difference in the visuo-graphomotor tests (TMT-A, and B, DSST, PST-C). For PST-D, RAVLT1, we do not have evidence strong enough for any claims. Means and standard deviations, the significance of difference (*p*-value), Eta-squared, and Bayes factors (BF_10_) are presented in Table 3.

**Table 3:** Cognitive performance of the groups (Means, Standard Deviations, or Confidence Intervals), the significance of the difference, effect size as Eta-squared, Bayes factor, and *Post hoc* analyses (*p*-value).

		N	Prague mean (SD/CI)	Towns mean (SD/CI)	Villages mean (SD/CI)	*p*-value	η^2^	BF_10_	PxT	PxV	TxV
Story		324	5.74 (1.12)	5.16 (1.25)	5.08 (1.32)	< 0.001	0.055	159.11	0.002	< 0.001	n.s.
TMT-A+		324	38.24 (29.13–50.10)	39.06 (27.93–54.46)	38.84 (28.42–52.95)	0.868	0.001	0.04	n.s.	n.s.	n.s.
TMT-B+	@	324	85.86 (60.05–122.58)	91.13 (62.44–132.78)	89.05 (62.09–127.53)	0.215	0.010	0.13	n.s.	n.s.	n.s.
DSST	@	324	56.16 (11.93)	53.37 (12.30)	54.26 (12.82)	0.103	0.014	0.27	n.s.	n.s.	n.s.
CS Visuo-Grapho	@	324	0.08 (0.81)	-0.07 (0.86)	-0.02 (0.86)	0.180	0.011	0.16	n.s.	n.s.	n.s.
PST-D	@	323	13.47 (2.45)	14.59 (4.56)	13.16 (3.20)	0.015	0.026	1.62	n.s.	n.s.	0.009
PST-W	@	323	16.52 (3.50)	18.34 (5.00)	16.51 (3.72)	0.002	0.039	13.75	0.004	n.s.	0.004
PST-C+	@	323	30.15 (22.30–40.66)	31.86 (23.81–42.52)	31.35 (22.19–44.12)	0.206	0.010	0.08	n.s.	n.s.	n.s.
VF Animals	@	324	25.67 (5.35)	23.07 (5.01)	24.10 (5.24)	< 0.001	0.048	22.56	0.001	n.s.	n.s.
RAVLT1		324	6.14 (1.96)	5.59 (1.75)	5.47 (1.50)	0.011	0.028	2.18	n.s.	0.015	n.s.
CS Verbal	@	323	0.16 (0.60)	-0.19 (0.77)	0.02 (0.70)	< 0.001	0.048	53.61	0.001	n.s.	n.s.
SRT+		316	398.24 (319.46–496.38)	475.53 (351.91–642.46)	448.71 (352.36–571.32)	< 0.001	0.084	7553.64	< 0.001	0.003	n.s.
GNG+		312	584.07 (501.49–680.22)	631.89 (534.22–747.37)	620.28 (526.33–730.96)	< 0.001	0.042	15.06	0.002	0.023	n.s.
CS Attention		312	0.34 (0.79)	-0.25 (1.00)	-0.08 (0.88)	< 0.001	0.071	1031.36	< 0.001	0.004	n.s.


*Values presented in the table are in standard, non-transformed units except for + scores, which were logarithmically transformed for the analyses (ln) and then back-transformed. CS: composite score. CI = confidence intervals (for logarithmical transformations). @ = ANCOVA with age as the covariate. η^2^ = eta squared – univariate ANOVA, effect size. BF_10_, Bayes Factor. P, Prague; T, Towns; V, Villages. PxT, PxV, TxV =* post hoc* analyses of differences between two types of environment, *p*-values. n.s. = non-significant, *p* > 0.01.*

Sidak’s *post hoc* analyses were conducted for the three pairs (Prague vs. towns; Prague vs. villages; and towns vs. villages). *Post hoc* pairwise comparisons (Table 3) showed that the differences are mostly driven by the Prague versus towns pair. Persons from towns and villages had fairly similar performance in most tests. The overall inspection of results revealed that the group from towns had low performance when compared to persons from villages or the city of Prague.

## Discussion

The goal of this study was to ascertain possible differences in cognitive performance in senior residents in three distinct types of settlement in the Czechia.

Analyses revealed that retired, young-old, adults not suffering from dementia who live the majority of their adult life in the capital city of Prague performed significantly better than residents of towns and villages in a test of verbal episodic memory (Story), in verbal timed tests (Prague Stroop Test and Category Verbal Fluency Test – Animals), and attention tests (Simple Reaction Time and Go/No-Go). We found no differences in scores of visuo-graphomotor timed tests (TMT and DSST).

The specific effect of the reported differences becomes obvious, for example, when we adapt the regression equation for the calculation of z-scores (standard deviation scores) in Category Verbal Fluency – Animals and (1) include only the usual predictors of age and education, or (2) add also the settlement type:

(1) Age- and education-included regression equation:

(1) $$z\;score_{1}=\frac{RS-(38.771-0.247*Age+1.542*Edu)}{5.299}$$

(2) Age-, education-, and settlement-included regression equation:

$$z\;score_{2}=\frac{RS-(40.312-0.276*Age+1.509*Edu+2.020*Pr-0.628*To)}{5.299}$$

RS, Raw score (number of named animals); Age (in years); Edu, level of education (1 = lower, 2 = higher); Pr, Prague (0 = no; 1 = yes), To, town (0 = no; 1 = yes).

For an illustration, Table 4 shows several examples of z-scores from raw scores obtained from older villagers.

**Table 4:** Examples of z-scores received from the regression equations.

Age	Education	Raw score	Prague	Town	z-score_1_	z-score_2_
74	1	11	0	0	-2.08	-1.96
69	1	15	0	0	-1.56	-1.47
74	2	15	0	0	-1.62	-1.49
73	2	13	0	0	-2.04	-1.92
72	1	17	0	0	-1.04	-0.93


The differences are not large, but in some cases, we can imagine that it could lead to different diagnostic conclusions if it passes a cut score, e.g., for a cognitive impairment. If 2 standard deviations (or 1,5 or 1 standard deviation; as those are used) are accepted as a decisive borderline for psychometrically diagnosed mild cognitive impairment, it would automatically lead to different conclusions (Arsenault-Lapierre et al., 2011; Bondi et al., 2014). On the other hand, at this moment, we cannot predict if the difference associated with settlement would result in different cut-scores and other parameters stemming from validation studies. Our results suggest that in the planning of test standardization studies, i.e., normative and validation studies of cognitive tests, settlement type should be incorporated and sample descriptions should contain information about the environment in terms of the level of urbanicity.

The city of Prague is proverbially hectic and perceived as such by older persons (Frydrychova et al., 2017). Our results suggest that the complex lived-in environment moderated the cognitive performance of its long-term dwellers regardless of how they subjectively perceived it. A parallel between perceived busyness and hectic pace of life seems natural. Accepting that parallel, our results are in line with findings of Festini et al. (2016), who showed that busier older persons tend to have better cognition, especially episodic memory. One can also imagine that life in a hectic environment of a city is associated with chronic mild stress, which was shown to promote declarative memory (Sandi, 2013), as was the case in our study.

Our findings indicate that life in the city stimulates verbal communication and attention (promptness of reaction to visual stimuli). An implication may be that cognitive performance reflects an adaptation to one’s environmental demands (Lawton and Nahemow, 1973). This seems to be in congruence with the “use it or lose it” hypothesis (Salthouse, 2006) and with Schaie’s and Schooler’s theories of environmental influences on cognitive functioning (Schaie, 1980; Schooler, 1984), which could be applied in this case as suggesting that time pressure in a city forces city dwellers to learn at a quicker pace when solving tasks. Additionally, there seems to be accord with the pace-of-life syndrome (POLS) hypothesis (Ricklefs and Wikelski, 2002; Dammhahn et al., 2018), positing that populations in certain ecological conditions share various traits that evolved in the particular conditions, and lately it has been extended to behavioral studies (Reale et al., 2010). Thus, it could be expected that persons sharing certain environments do develop similar patterns of cognition. In the city, there is denser traffic and a higher density of population. Personal interactions with these factors are highly individual, and it is uncertain whether we can make general assumptions. Nevertheless, we propose that the more complex environment of a city has, in the long term, a positive impact on verbal production and faster reaction time to stimuli in its older inhabitants. This is in accord with a recent review of environmental influences on cognitive aging (Cassarino and Setti, 2015), positing that attention and executive function are most affected by the environment. Both in this and the previous NANOK study, Praguers had superior performance in verbal tests (especially episodic memory and category verbal fluency). Besides the above mentioned, we think of an effect of social interactions. While one can be lonely in the city, in general it is possible that the higher density of population, and necessary interactions in public transport means, etc., do support verbal abilities. McHugh Power et al. (2019) suggested that good memory, measured with a word-list test and category verbal fluency, suppressed the negative impact of loneliness on social engagement. We may hypothesize that social engagement promotes verbal memory in adulthood.

Surprisingly, *post hoc* analyses revealed the lowest performance in cognitive tests in towns, rather than in villages. As there were no differences in the most commonly monitored features between our groups, the driving factors may either be other hidden characteristics of our groups for which we did not control, or it could be that somehow the life in villages is more stimulating than in smaller towns. For example, it could be that villagers need to drive cars more than inhabitants of towns, or that transportation in the city is dense. Both city and village environments may pose greater demands on attention than in small towns, where there are not such significant distances to pass between destinations, and many locations are within walking distance and the traffic is less dense than in the city. It could be that life in towns is indeed the most comfortable and the least cognitively demanding: distances to commute are usually the shortest and there may be a comfortable level of socialization in the community (people know each other and meet acquaintances quite often in the streets, but it is not as intimate as it is in villages). Alternatively, it is possible that various towns are more heterogeneous in terms of the shared lifestyle of most inhabitants. However, we do not have the data to test this hypothesis.

The differences we identified between the settlement groups could be driven by other characteristics besides the settlement and shared environment. They could be a function of the sample, as in any research. Thus, we tried to draw out relevant characteristics in the context, as described below.

Education and mentally challenging activities, including profession and leisure time, are considered a part of the cognitive reserve and are stipulated to have a protective effect on cognition in older age (Scarmeas and Stern, 2003; Hertzog et al., 2008; Stern, 2009; Fisher et al., 2014; Lojo-Seoane et al., 2014; Nyberg and Pudas, 2019). The important protective role of educational attainment against pronounced cognitive impairment was reported by Weden et al. (2018) in a recent study of rural-urban disparities in the United States. While our results may appear logical, because one can expect that in a city with governmental bodies, research institutes, and universities, there are more highly educated residents, and education is a known predictor of cognitive performance (Mungas et al., 2009), in this case, the education level in the groups do not differ, and thus the ratio of highly educated persons is not the cause of the findings.

We previously showed that leisure activities, especially computer-use and doing crossword puzzles or quizzes mitigated the effect of lower education on cognitive performance in COURAGE (Frydrychova et al., 2018). van der Meer (2008) concluded that the diversity of leisure activities, in which older people are involved, is not associated with their intrapersonal characteristic, but it is influenced by the lived-in environment. Our results are in accord with this as there are significant differences in specific activities between three types of settlements. Despite the fact, that there are similar opportunities for leisure activities including attendance of U3A courses in older age in all Czechia regions even if they may differ in some aspects (some regions are more agricultural than others) (Vidovicova, 2018). In our study, retired Praguers engage more often in computer-use and U3A or other courses than senior non-Praguers. On the other hand, villagers report more non-specific physical exercise including gardening than persons from towns or Prague. It is possible that those types of activities are finally the driving force to the found differences in cognitive performance between our sub-samples. However, our study was not planned to investigate lifestyle profoundly. It was designed to find whether there are differences in cognitive performance between dwellers of the specified types of settlements and help to aim further research. A sum of activities used in our study is a simplified method that points to a direction of interest. Lifestyle and specific activities need to be researched further in the context of settlement types, and cognition: what is it exactly that causes those differences?

It was reported that older persons living in urban areas have more depressive symptoms than residents of rural areas in Iowa, United States (Evans, 2009) or Britain (Walters et al., 2004), but this was not the case in our study. The groups from Prague and other settlements did not differ in reports of depressive symptoms, nor in the use of psychopharmacological medication. That could be due to our recruitment strategy, which included the young-old (generally healthy) population. As depression is considered a risk factor or a prodromal symptom for cognitive impairment in older age (Weisenbach et al., 2012), we controlled for these symptoms, and also for other potentially negative factors, such as psychopharmacological treatment, subjective poor health (Georgi et al., 2018), stress due to negative life events, and personal economy. The groups did not differ in any of these factors and there was no significant association between impeding factors and cognitive performance.

The aforementioned similarities in the characteristics of the groups lead us to believe the groups were comparable, and that the differences identified in terms of cognitive performance are well supported by our data.

Our results do not completely agree with the pilot data from the NANOK study. In the pilot, TMT, PST, and VF Animals (but not verbal memory tests: Digit Span and Philadelphia Verbal Learning Test) showed a significant difference between citizens of Prague and non-citizens of Prague. Our pilot conclusion was that the overall picture showed the greatest effect of the current location of living on timed tests of executive function (Horáková et al., 2016). However, the present study offers another picture. There is a consistent effect of place of residence on verbal and attention tests, and none in visuo-graphomotor tests. This discrepancy could be due to a more lenient recruitment in NANOK, as the sample included persons of a wider age range (60–92 years old, mean age 77.7 years), irrespective of their employment or retirement, and the stratification was based only on the current place of residence, not on their long-term place of residence (some Czechia older persons move to the countryside to their previous “summer houses” in their retirement years, and thus may dramatically change their lived-in environment). The COURAGE sample was younger than that of NANOK (COURAGE: only young-old adults, 60–74 years of age, retired for a minimum of 2 years, but for a significantly shorter period than the NANOK sample, etc.) and was carefully selected respecting comprehensive criteria and long-term place of residence and work. Thus, the data are not fully comparable. Nevertheless, despite the differences in the samples, there is a consistency in the found effect of urban residence on timed verbal tests (VF Animals, Prague Stroop test) and absence of an effect on DSST.

Prague was the only urban settlement with a population exceeding 50 thousand in our study, had the densest network of public transportation enabling easy mobility, and offered the most extensive variety of educational and cultural events for retired persons. We propose that our findings may be adequate for other European countries, where many cities are similar to Prague and smaller towns and villages to those in Czechia on the key parameters.

The generalizability of our results especially to old- and oldest-old people is questionable. One aspect is related to cognitive trajectories, with an often-referred-to inflection point in 75 years of age (Schaie and Willis, 2010), and neuroplasticity better preserved in young-old than in old-old persons (Borella et al., 2014; Navarro and Calero, 2018). Those facts would make it too optimistic to apply the findings to the next developmental stage. Also, we need to bear in mind that old- and oldest-old people are survivors of World War II (or even WWI) and political upheavals in the socialist era. The tumultuous years in the majority of the 20th century provided quite different conditions for people to attain higher education. Gender roles played more critical roles in the availability of training and profession, too, which resulted in an entirely different picture of gender ratios in jobs in various age cohorts (CZSO, 2016). Both education and profession are known to correlate to some extents with cognitive performance. Therefore, we suggest that our findings are rather cohort-specific, and their validity in other age cohorts needs to be researched. We also propose that similar age-cohort effects could be observed in European countries, as fundamental historic moments in the last century, not in detail but at large, are shared there.

### Limitations

The study has several limitations. Self-selection and snowball sampling lead to the usual problem of generalizability of the findings. Nevertheless, the recruitment strategy was the same in all three types of settlement, and therefore, we expect the observed trends to correspond to the general ones. Due to time-consuming tests presented on tablets, adding more measures would have made the overall assessment too lengthy, and thus we operate with limited data. Below, we offer several avenues for further research in the context of culture-typical environments.

While our study was designed to ascertain whether there are differences in timed cognitive tests depending on a long-term settlement, and the three groups are balanced in many important features such as gender, education, and number of activities performed in adulthood and at present, etc., there are aspects that are out of our control. Among those, engagement in activities may differ substantially even if the simple report is the same. A study focused on different types of activities, as well as intensity and number of years devoted to them, would be desirable, although this would need to be realized in sequential sessions to prevent fatigue in the participants. The unexpected result of the lowest performance in most tests in town residents points to the necessity to focus in more detail on the characteristics of that type of environment. Our study data do not contain information on further characteristics of particular settlements. A closely related limitation is a lack of objective data about the amount of perceptual information, and a lack of data on the use of the environment, or subjective environmental preference. These issues are pointed out by Cassarino and Setti (2016) as relevant in studies of individual interaction with their surroundings. In our study, the groups did not differ in how they perceived their pace of life during an economically productive adulthood, i.e., how hectic it was for them and how much time pressure they were subjectively dealing with. There was no difference in terms of numbers of activities during productive years or currently in retirement, nor in the type of profession (manual vs. mental). That does not inform us about the objective complexity of the environment, but it does concern their subjective feeling of time pressure.

Another interesting aspect that we are not able to fully document in this study is social engagement. Social engagement may play a very similar role to cognitive engagement throughout life, as findings from other studies suggest, yet a consensus has not been reached (Weber, 2016). For example, Krueger et al. (2009) confirmed that the frequency of social activity and the level of perceived social support are correlated with better cognitive performance, and higher social engagement in older persons was associated with a decreased risk of dementia (Zhou et al., 2018). An experimental study showed a similar cognition-enhancing effect of social engagement or cognitive training (Stine-Morrow et al., 2014). Håkansson et al. (2009) and Feng et al. (2014) analyzed marital status and cognition and found that being married is associated with a decreased risk of cognitive impairment in later age. Our data only provide information on having a partner and living alone, which show differences between the groups, but do not cross the point of significance concerning cognitive performance. In our sample, we observed the highest prevalence of people who had a partner in villages compared to towns or Prague; and lowest in towns. Thus, we could hypothesize that having a partner, which requires regular communication, may have a cognition-enhancing effect. Or the stress caused by losing a partner could have a detrimental effect. This effect may be partly responsible for the generally lowest cognitive performance in our subsample from towns. A synergy of partnership, stimulating leisure activities and environment may result in the observed differences in cognition. However, we could not detect significant direct association of partnership with cognition. Data on social networks, domains of social engagement, and subjective loneliness of our participants are not available. Future studies could relate those aspects and lifestyles in various types of settlements.

While we have some information related to health (e.g., included in the impediments), and the inclusion criteria comprised some medical history conditions, we are aware that all participants have an individual health history and health behaviors throughout their lives. These, of course, could affect their cognitive performance. The “nurture” aspect had a long time to influence each older person in many ways. It is thus impossible to obtain a totally homogenous sample in every aspect of life history. However, our effort was to reach as much relevant homogeneity as possible, as documented in Table 1.

Another shortcoming is an inclusion of the traditional majority Czechia Caucasian persons only, while the numbers of minorities living in Czechia have risen since the Velvet Revolution in 1989 (CZSO, 2014). There are many people of Romani ethnicity (about 240 thousand; i.e., 2.2%) that may or may not declare themselves as Roma (Government of the Czech Republic, 2018), who form a unique society with a specific historical culture, and who were discriminated against in the past, including factual inaccessibility to higher education in Czechia (Miskovic, 2013, p. 36). Other ethnic or nationality groups, such as the Vietnamese and Ukrainians, among others, live and work here. They are second- or third-generation Czechia inhabitants, or first-generation newcomers. In any case, the older persons do not share a common environment, and culture with the majority population. Therefore, our work may be relevant only for the traditional majority population in Czechia.

Our study is cross-sectional, and our data do not substantiate an inference on long-term associations into the very old age, and therefore cannot have an ambition to point at preventive interventions. Nevertheless, our study was designed to ascertain differences or similarities of cognitive performance in older persons, especially in the context of normative studies and subsequent clinical assessment.

The influence of various preventive or harmful factors on aging and cognition have a long future of research ahead.

Settlement type or lived-in environment is significantly associated with performance in standard cognitive tests in older persons. Therefore, it would be most helpful if future psychometric, normative, and validation studies listed where their samples were drawn from in terms of the types of settlements. So far, this information is either completely omitted or is only vaguely described. Such information could help clinicians in their evaluation of results near cut-scores and avoid errors in diagnosis, especially false-positive errors (Mungas et al., 2009).

## Conclusion

The purpose of this study was to find further evidence on the relation of the urban environment and cognitive performance in older age and suggest implications for practice. Our findings should motivate authors of normative and validation studies of neuropsychological methods to include a more detailed description of their samples as to the environment from which their subjects come. If there are substantial differences between lifestyles between rural and urban environments, they should consider different datasets for such environments. For clinical practice, our suggestion is to consider whether the available norms correspond, besides age, gender, and education, to the type of settlement of the patient in applicable cases. This should help to achieve culturally adequate procedures in cognitive assessment (Rivera Mindt et al., 2010).

## Data Availability

The project was registered at Open Science Framework, and the data were made available there: https://osf.io/cmt72/.

## Ethics Statement

This study was carried out in accordance with the recommendations of the Institutional Ethical Review Board of the National Institute of Mental Health with written informed consent from all subjects. All participants signed informed consent forms in accordance with the Declaration of Helsinki. The study protocol was approved by the Institutional Ethical Review Board of the National Institute of Mental Health, Klecany, Czechia (under Nr. 61/16).

## Author Contributions

HSG contributed to the conceptualization and funding acquisition, investigation, data curation, and overall writing of the manuscript. ZF contributed to the investigation and data curation, data analyses, organization and editing of the manuscript. KHV contributed to the conceptualization and funding acquisition, and editing of the manuscript. LV contributed to the editing of the manuscript. ZS contributed to the data analyses and editing of the manuscript. JL programmed the software used in the project, and contributed to the data analyses, organization and editing of the manuscript.

## Conflict of Interest Statement

The authors declare that the research was conducted in the absence of any commercial or financial relationships that could be construed as a potential conflict of interest.
